# The use of internet-based smartphone apps consistently improved consumers' healthy eating behaviors: a systematic review of randomized controlled trials

**DOI:** 10.3389/fdgth.2024.1282570

**Published:** 2024-01-12

**Authors:** Awole Seid, Desta Dugassa Fufa, Zebenay Workneh Bitew

**Affiliations:** ^1^ Department of Adult Health Nursing, College of Medicine and Health Sciences, Bahir Dar University, Bahir Dar, Ethiopia; ^2^ Center for Food Science and Nutrition, Addis Ababa University, Addis Ababa, Ethiopia; ^3^ Haramaya Institute of Technology, Haramaya University, Dire Dawa, Ethiopia; ^4^ Saint Paul’s Hospital Millennium Medical College, Addis Ababa, Ethiopia

**Keywords:** smartphone apps, healthy eating behavior, systematic review, RCTs, web based applications

## Abstract

**Introduction:**

Digital tools, such as mobile apps and the Internet, are being increasingly used to promote healthy eating habits. However, there has been inconsistent reporting on the effectiveness of smartphones and web-based apps in influencing dietary behaviors. Moreover, previous reviews have been limited in scope, either by focusing on a specific population group or by being outdated. Therefore, the purpose of this review is to investigate the impacts of smartphone- and web-based dietary interventions on promoting healthy eating behaviors worldwide.

**Methods:**

A systematic literature search of randomized controlled trials was conducted using databases such as Google Scholar, PubMed, Global Health, Informit, Web of Science, and CINAHL (EBSCO). The Preferred Reporting Items for Systematic Reviews and Meta-Analyses (PRISMA) guidelines were followed to prepare the entire document. EndNote (version 20) was used for reference management. The risk of bias in the articles was assessed using the “Revised Cochrane Risk of Bias tool for randomized trials (RoB 2.0)” by the Cochrane Collaboration. Narrative synthesis, using text and tables, was used to present the results. The study was registered in PROSPERO under protocol number CRD42023464315.

**Results:**

This review analyzed a total of 39 articles, which consisted of 25 smartphone-based apps and 14 web-based apps. The studies involved a total of 14,966 participants. Out of the 25 studies, 13 (52%) showed that offline-capable smartphone apps are successful in promoting healthier eating habits. The impact of smartphone apps on healthy adults has been inconsistently reported. However, studies have shown their effectiveness in chronically ill patients. Likewise, internet-based mobile apps, such as social media or nutrition-specific apps, have been found to effectively promote healthy eating behaviors. These findings were consistent across 14 studies, which included healthy adults, overweight or obese adults, chronically ill patients, and pregnant mothers.

**Conclusion:**

Overall, the findings suggest that smartphone apps contribute to improving healthy eating behaviors. Both nutrition-specific and social media-based mobile apps consistently prove effective in promoting long-term healthy eating habits. Therefore, policymakers in the food system should consider harnessing the potential of internet-based mobile apps and social media platforms to foster sustainable healthy eating behaviors.

## Introduction

The concept of digital access goes beyond simply having physical access to the Internet. It also includes the ability to search, evaluate, organize, and perform tasks using digital devices in various aspects of life, such as learning, work, and social interactions. The United Nations, through its Sustainable Development Goals (SDG 9), aims to improve digital literacy by setting targets to achieve universal and affordable internet access by 2030 (1, 2). Although more than 80% of people in developed countries use the Internet, 32% of Europeans lack basic digital skills (3–5). This indicates a significant gap in digital literacy within developed nations, and the adoption of digital technology is influenced by factors such as age, residence, socioeconomic status, education level, and others (6).

Digital technologies, like mobile health apps (mHealth), can be used for public health dietary education, promotion, and empowering individuals to take care of themselves (7). It has been indicated that advocating for people-centered health systems enabled by digital health is a strategic objective of WHO's global strategy on digital health 2020–2025 (8). Hence, to achieve this strategic objective, it is imperative to improve digital health literacy at the population level. This entails addressing attitudes, practices, and public awareness of digital health (8).

Unhealthy eating increases the risk of chronic diseases. However, many current strategies for promoting healthy eating are not sustainable in the long term (9). As a result, mobile apps are being used more frequently to deliver behavioral health interventions. These digital platforms can help people improve their food behavior over a longer period of time (10, 11). According to studies, digital tools such as mobile apps, the internet, and video games can promote healthy eating (12–14). Digital health technology is also receiving policy investment due to its ability to deliver healthcare to more people in a cost-effective manner.

One study indicated that the increase in lifestyle diseases, which are often linked to poor consumer food behavior, necessitates the need for accessible and efficient digital solutions (15). Although digital technologies (DTs) have been used to raise awareness and encourage the consumption of healthy foods, their effectiveness has not been consistently documented (16, 17). Furthermore, upon closer appraisal of previous reviews reveals certain limitations. These limitations include a limited number of included studies (18), studies conducted on a single population group (19–23), examination of different types of DTs together (24), the inclusion of articles conducted in a single country (25), the use of DT assessment to measure dietary intake instead of intervention (26), exclusion of recently published articles (9, 27, 28), and lack of assessment of specific interventional strategies, such as an artificial intelligence chatbot (29). It is important to note that the use of digital technology, especially smartphone apps, is still in an exploratory phase (30). Therefore, a comprehensive systematic review is needed to address a diverse population, cover various study periods, and include rigorous quality assessment. Lastly, this review aims to offer robust recommendations for practice, policy, and future research.

### Review question

• What is the effect of smartphone apps and web-based dietary interventions on healthy eating behaviors?

The scope of the study and eligibility criteria were defined using the Population, Intervention, Comparison, Outcomes, and Study (PICOS) framework as follows, before conducting the article search (31).

• Population (P): Healthy adults, overweight adults, obese adults, children, adolescents, chronically ill patients, pregnant women, and breastfeeding mothers. Studies from all countries, regardless of their income level and development, were included.
• Intervention (I): Smartphone app-based dietary education, whether nutrition-specific or through social media, includes counseling for improving dietary adherence, maternal diet counseling, guidance on exclusive breastfeeding and complementary feeding, promoting the consumption of fortified foods and a diverse diet, counseling on healthy and sick child feeding, and any other intervention aimed at improving healthy dietary practices in the general public.
• Comparison (C): No digital technologies, as well as similar dietary interventions delivered through other digital technologies such as web-based platforms, phone calls, and text messages.
• Outcome (O): Healthy eating behaviors/practices:
  - Increase consumption of fruits, vegetables, legumes (such as lentils and beans), nuts, and whole grains (such as unprocessed maize, millet, oats, wheat, and brown rice).
  - Avoid or limit table sugar to less than 5% of total energy intake.
  - Decrease salt intake to less than 5 g per day.
  - Practice exclusive breastfeeding for the first 6 months of life.
  - Introduce complementary feeding.
  - Breastfeed optimally up to 2 years of age.
  - Reduce saturated fats to less than 10% of total energy intake.
  - Avoid industrially-produced trans-fats.
  - Follow special diets like DASH and Mediterranean diet.
  - Read nutrition labels.
  - Prevent food contamination.
  - Make mindful food choices.
  - Make informed food purchases.
  - Consume fortified foods, including the use of multiple micronutrient powders at home.
• Time: No time restriction.
• Language: Articles with abstracts written in English were included for articles retrieved from PubMed, while articles from other data sources were searched without language restrictions.
• Types of included studies: Randomized controlled trials (RCTs) that reported relevant outcomes of interest were included. However, systematic reviews and meta-analyses, observational studies, unpublished studies such as theses and dissertations, editorial comments, non-human studies, conference proceedings, case reports, case series, and duplicate publications using similar data were excluded from the current study.

## Materials and methods

### Data sources and search strategies

This document has been prepared based on the Preferred Reporting Items for Systematic Reviews and Meta-Analyses (PRISMA) guidelines (32). In this study, articles published up to June 2022 were included. To retrieve articles, reputable database sources were explored independently by three authors. The following databases were explored comprehensively: PubMed (including Medline), Scopus, Web of Sciences, Embase, Global Health, Cumulative Index to Nursing and Allied Health Literature (CINAHL) (EBSCO), WHO's Institutional Repository for Information Sharing (IRIS), Informit Health Collection, Food Science and Technology Abstracts (FSTA) (EBSCO), and references of previously published reviews (snowball technique). Advanced searching was employed during the search process for those databases. The retrieved articles were exported to EndNote version 20 software, so the removal of duplicates and citations was performed using this software. Key terms were verified for appropriateness before the actual search. For instance, the following search strings were employed to search articles from PubMed (Table 1).

**Table 1:** Search terms used to access published studies in the pubMed database, 2022.

Database	Search terms	Number of articles
PubMed	"Digital Technology"[MeSH Terms] OR ((((“Mobile Applications"[MeSH Terms] OR “Internet-Based Intervention"[MeSH Terms]) AND (“Diet"[MeSH Terms] OR “diet, ketogenic"[MeSH Terms] OR (“diet, gluten free"[MeSH Terms] OR “diet, carbohydrate restricted"[MeSH Terms] OR “diet, mediterranean"[MeSH Terms] OR “diet, protein restricted"[MeSH Terms] OR “diet, fat restricted"[MeSH Terms] OR “diet, macrobiotic"[MeSH Terms] OR “diet, vegetarian"[MeSH Terms] OR “diet, sodium restricted"[MeSH Terms] OR “diet, reducing"[MeSH Terms] OR “Diet Therapy"[MeSH Terms]))) OR “diet, healthy"[MeSH Terms] OR “Feeding Behavior"[MeSH Terms]) AND (“2013/08/16 00:00″:"3000/01/01 05:00"[Date—Publication] AND “randomized controlled trial"[Publication Type]))	5,837

### Data extraction procedure

The articles in this systematic review were assessed and selected by the three authors (ZWB, AS, and DD). The data extraction sheet was created using Microsoft Excel. The sheet includes authors' names, publication years, research titles, study settings and designs, study populations, sample sizes, types of smartphone apps, types of dietary interventions, assessment methods for outcome variables, duration of interventions or follow-ups, methods of intervention, main findings, and limitations. Any discrepancies during the extraction process were resolved through discussion among the authors.

### Quality assessment of studies

In this study, the Cochrane Risk of Bias Assessment Tool Version 2 (RoB 2) was used to evaluate the quality of articles. The tool consists of components such as yes (Y), probably yes (PY), no (N), probably no (PN), and no information (NI). Bias can arise from various factors, including the randomization process, deviations from intended interventions, missing outcome data, measurement bias of the outcome, and selection bias in reporting results. The overall risk of bias is determined by three components: low risk, high risk, and some concerns. If all five components or domains are assessed as low risk, the overall risk of bias is classified as “low risk.” If one of the components has some concerns, the overall risk of bias is categorized as “some concerns.” On the other hand, if any of the five components are assessed as high risk or if any of the two domains have some concerns, the overall risk of bias is classified as “high risk” (33). The qualitative presentation of the overall risk of bias is represented by a visual graph with green, yellow, and red colors, indicating low risk, some concerns, and high risk, respectively**.**

### Data synthesis

The results of the study are presented through text and tables. Since the effect measures reported in the study are varied, a quantitative summary or meta-analysis was not conducted. Furthermore, the results are summarized based on the following subgroups: type of smartphone apps, study population, type of public health dietary interventions, duration of intervention, and country income classification. To provide a narrative synthesis, vote counting and the direction of the effect were utilized (34). The findings are displayed using summary tables.

## Results

### Literature search and study selection

**Figure 1:**
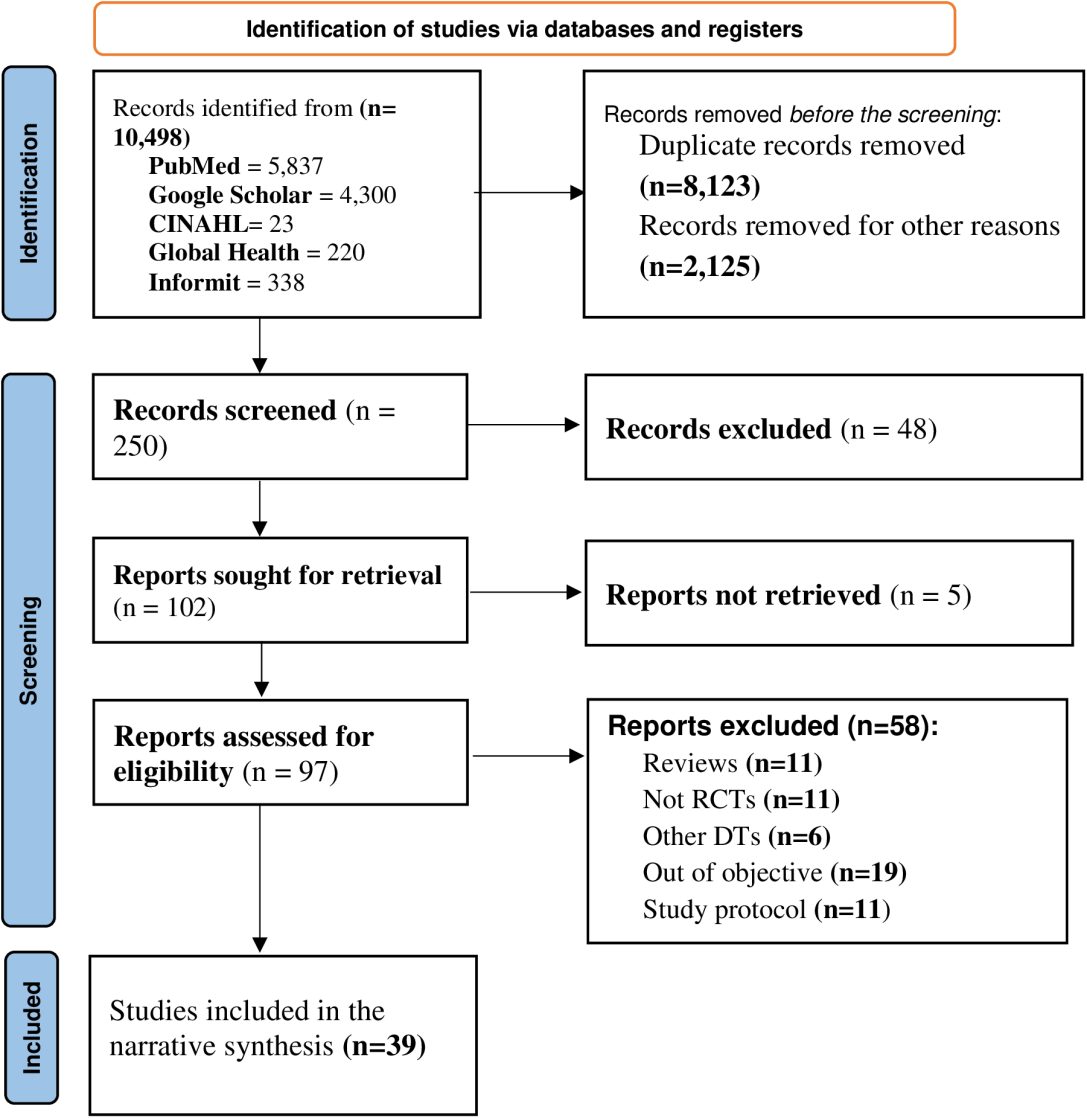
Study selection proces using the PRISMA flowchart, 2022.

The search identified a total of 10,498 electronic records. After removing 10,248 records due to duplication and irrelevancy, 250 titles were screened for potential eligibility. Following the removal of 48 articles that were unrelated and five articles that could not be retrieved, a total of 58 articles remained. Among these, 11 were excluded as they were not randomized controlled trials (32, 35–44), 11 were reviews (18–22, 24, 26, 27, 29, 31, 45), six studies used other types of digital technologies (DTs) (46–51), 19 studies did not align with the review objective (52–70), and 11 studies were study protocols (71–76). Finally, a total of 39 studies were included in the review, of which 25 utilized smartphone apps that could function both online and offline, and the remaining 14 used web-based apps that required an internet connection to function (Figure 1).

### Characteristics of the included articles

All 39 articles included in this systematic review were randomized controlled trials. The majority of the studies (31) were reported from high-income countries, while the remaining nine studies were reported from middle-income countries. Similarly, 16 of the studies were reported from European countries, 10 from the USA, and the remaining studies were reported from Asian countries. All of the included studies were published between 2011 and 2022. The sample sizes ranged from 24 to 1,859 participants. Regarding the study population, 12 studies were carried out in healthy adults, seven in overweight/obese adults, nine in chronically ill patients, six in pregnant mothers, and five in children. In addition, the duration of the intervention ranges from 10 days to 24 months. Regarding the type of interventions, 20 studies focused on comprehensive dietary interventions, nine studies targeted fruit and vegetable consumption, five studies focused on limiting salt or sodium intake, three studies focused on adherence to the Dietary Approaches to Stop Hypertension (DASH) and Mediterranean diets, two studies focused on promoting exclusive breastfeeding, and one study examined consumers' food choices.

### Risk of bias of included studies

**Figure 2:**
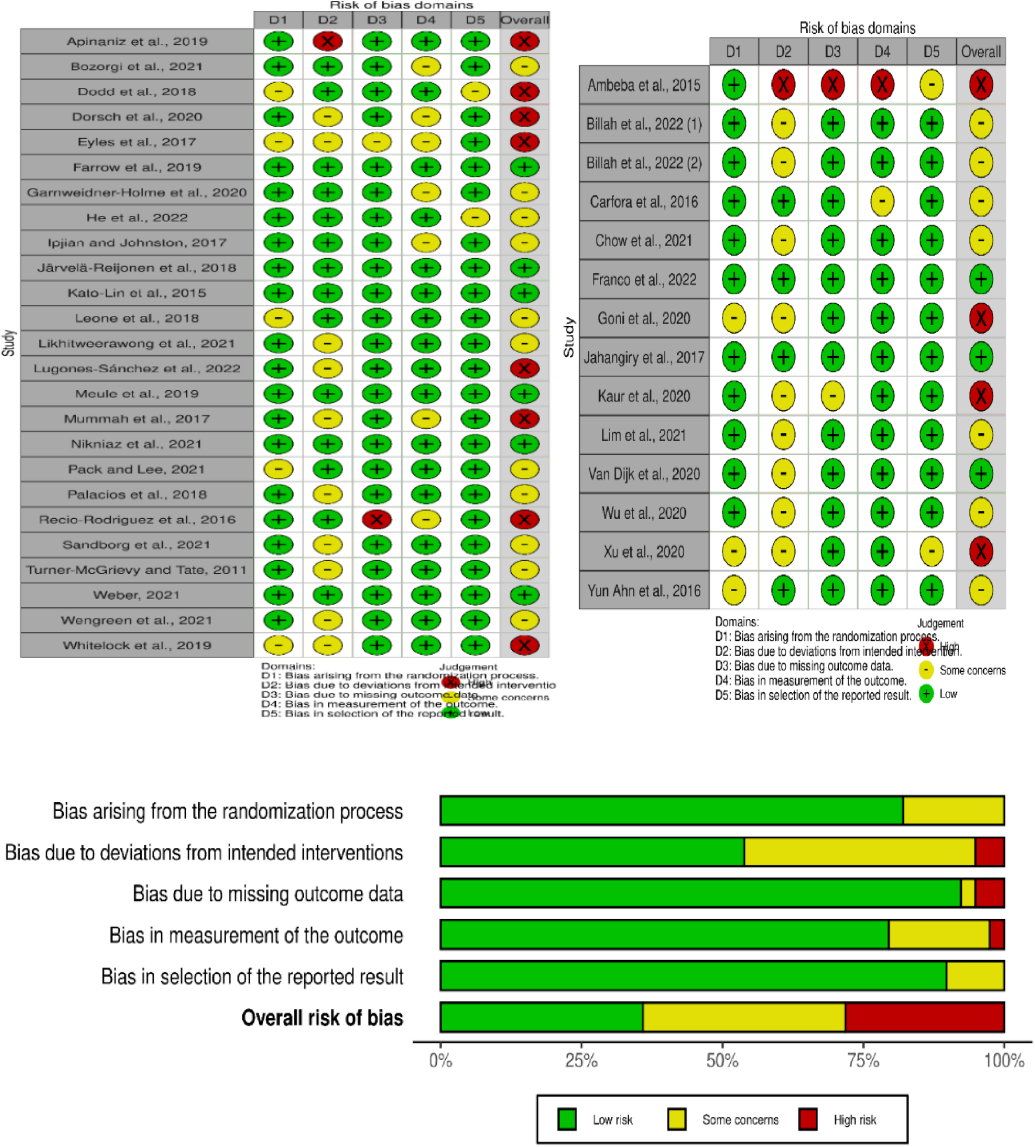
The overall risk of bias of included studies assessed with the cochrane risk of bias tool, 2022.

After the quality of studies was evaluated using Cochrane RoB 2, nine studies were identified as low-risk, 18 studies had some concerns, and 12 studies had a high risk of bias. This shows that the results should be interpreted cautiously (Figure 2).

### Effectiveness of interventions

The data is categorized into smartphone apps and web applications for better understanding. Smartphone apps are designed for mobile devices like smartphones and tablets. They can be accessed without an internet connection once installed. On the other hand, web apps are designed to run on web browsers across different devices, including desktops, laptops, and mobile devices. Web apps require an internet connection to access since they are hosted on remote servers and accessed through web browsers.

### The effect of smartphone app-based interventions on healthy eating habits

Out of the 25 studies, 13 (52%) found that mobile apps encouraged healthier eating habits. The remaining 12 (48%) had mixed or no significant results. Smartphone app-based interventions were effective in promoting healthier eating habits among patients with chronic diseases. Only four studies involving healthy adults, two studies on children, and one study on pregnant women showed a significant change in eating behavior through smartphone apps.

The use of mobile apps to reduce sodium intake has been studied in various populations. Several studies, including Dorsch et al. (77), Eyles et al. (78), Hele et al. (74), He, et al. (79), Ipjian and Johnston (80) evaluated the effects of mobile apps such as LowSalt4Life, SaltSwitch, and MyFitnessPal on salt intake among different groups, including hypertensive patients, cardiovascular patients, schoolchildren and families, and healthy adults. These studies followed participants for periods ranging from 4 weeks to 12 months and found a significant reduction in dietary sodium intake as well as household purchases of salt. Moreover, the findings indicated that mobile apps were effective in providing salt restriction advice, even in restaurant and beverage settings.

Mobile apps have been shown to benefit adherence to the DASH diet and increase dietary self-efficacy among patients with hypertension and chronic kidney disease. A study conducted by Bozorgi et al. on 120 hypertensive patients over 8 weeks demonstrated that the use of smartphone apps improved adherence to the DASH and low-fat diets (81). In a study by Pack and Lee with 75 patients undergoing hemodialysis, a 30-minute face-to-face and online counseling program significantly increased the dietary self-efficacy of the patients (82).

The impact of smartphone apps on the eating behaviors of both healthy and overweight adults has been inconsistent. For example, Järvelä-Reijonen et al. conducted a study involving 219 overweight adults, which showed that a mobile app-based intervention delivered in a group session had a beneficial effect on improving dietary behavior (69). Similarly, Mummah et al. found that the use of a mobile app called “Vegatone” led to an improvement in daily vegetable consumption among overweight adults (83). In contrast to the previous results, several studies conducted on both healthy and overweight adults, with different sample sizes and study settings, did not find a significant impact on eating behavior. For example, a study by Kato Lin et al. involving 425 healthy adults in the USA found no improvement in users' engagement in tracking dietary patterns (84). Similarly, in Spain, a study conducted by Recio-Rodriguez et al*.* with 833 healthy adults showed no difference in adherence to the Mediterranean diet in the intervention groups (85). Likewise, a study by Meule et al. indicated that a smartphone-based approach did not affect the eating behaviors of adolescents (86). Further studies are needed to determine the cause of these disparate results.

Game-based mobile apps have been shown to significantly improve children's eating behaviors. A 3-month follow-up study by Wengreen et al. involved 1,859 children. The study found that presenting the smartphone app (FIT Game) as daily comic-book-formatted episodes, projected onto a large screen in the school cafeteria during lunchtime, led to an increase in the consumption of fruits and vegetables (87). Another study conducted by Farrow et al. involved 74 children between the ages of 3 and 6. The study revealed that playing a game-based mobile app called “Vegetable Maths Masters” led to an increase in both the preference for and consumption of vegetables (88) (Table 2).

**Table 2:** Summary of randomized controlled studies on the effect of smartphone App-based interventions on healthy eating behaviors, 2022.

S. N	Author (s), year	Study setting	Study population	Sample size	Outcome variable measurement	Follow-up period	Main finding
1.	Apinaniz et al., 2019 (89).	Spain	Obese and overweight patients	110	Adherence to dietary intake of fruit and veg at least 400 g /day	8 months	No significant differences in adherence to diet in the intervention group.
2.	Bozorgi et al., 2021 (81)	Iran	Hypertensive adults	120	Adherence to the DASH diet	8 weeks	mHealth was effective in the patient's adherence to treatment, DASH, and a low-fat diet.
3.	Dodd et al., 2018 (90)	Australia	Pregnant women with BMI >18.5 kg/m2	162	Healthy Eating Index (HEI)	28 weeks	The use of a smartphone app has no additional benefit in improving the HEI score
4.	Dorsch et al., 2020 (77)	USA	Hypertensive adults	50	24-hour urinary sodium excretion + FFQ	8 weeks	Mobile app intervention resulted in a greater reduction in the dietary sodium intake in adults with hypertension
5.	Eyles et al., 2017 (78)	New Zealand	People with cardiovascular disease	66	A random (spot) urine sample for sodium	4 weeks	A significant reduction in mean household purchases of salt was observed.
6.	Farrow et al., 2019 (88)	UK	Children aged 3–6 years	74	6-item food fussiness scale	2 weeks	The game app significantly increased the liking and consumption of vegetables
7.	Garnweidner-Holme et al., 2020 (91)	Norway	Women with Gestational Diabetes Mellitus	238	A healthy dietary score for Pregnant + FFQ	36 weeks	The use of mobile apps did not have a significant effect on healthy dietary scores.
8.	He et al., 2022 (79)	China	Schoolchildren and their families	592 children and 1,184 adults	24-hr urinary sodium excretion	12 months	App-based education programs using a child-to-parent approach were effective in lowering salt intake in adults but the effects were not significant in children.
9.	Ipjian and Johnston, 2017 (80)	USA	Healthy adults	30	24-hr urine sodium excretion	4 weeks	Smartphone apps facilitate the implementation of dietary advice like salt restriction.
10.	Järvelä-Reijonen et al., 2018 (69)	Finland	Overweight and obese adults	219	Questionnaires of eating behavior (IES-1, TFEQ-R18), diet quality (IDQ), and 48-hr dietary recall.	36 weeks	Mobile apps had a positive effect on eating behavior but did not affect diet.
11.	Kato-Lin et al., 2015 (84)	USA	Healthy adults	425	Healthy eating behavior index	4 months	Mobile based visual diary did not improve users’ engagement in dietary tracking compared to web-based non-visual diary
12.	Leone et al., 2018 (92)	USA	Lower-income communities	142	10-item National Cancer Institute F&V screener,	6 months	Intervention participants did not show significant improvements in perceived access to fresh fruit and vegetable.
13.	Likhitweerawong et al., 2021 (93)	Thailand	Children and adolescents with obesity	77	Healthy eating behavior questionnaire	6 months	There was no difference in participants’ engagement of healthy eating behaviors among the intervention groups.
14.	Lugones-Sánchez et al., 2022 (94)	Spain	Healthy adults with overweight and obese	650	Diet composition using FFQ	12 months	The intervention group reduced the intake of cholesterol and full-fat and increased the intake of whole-meal bread and whole-grain cereals.
15.	Meule et al., 2019 (86)	Austria	Adolescents	105	Food cravings questionnaire (FCQ-S), Dutch Behavior Questionnaire's.	10 days	A smartphone-based approach did not affect eating-related behaviors.
16.	Mummah et al., 2017 (83)	UK	Overweight adults	135	Daily vegetables servings, measured by an adapted Harvard FFQ	12 months	The intervention group significantly increased the daily of consumption vegetables
17.	Nikniaz et al., 2021 (95)	Iran	People with celiac disease	60	Knowledge questionnaire and the validated celiac disease adherence test (CDAT).	3 months	The mean post-intervention score of knowledge about gluten-free foods was significantly higher in the intervention groups.
18.	Pack and Lee, 2021 (82)	Korea	Hemodialysis patients	75	Serum phosphorus, potassium and albumin, self-efficacy and quality of life	8 weeks	Online counselling through a smartphone enables patients to identify real-time issues with eating habits and manage their diet by themselves.
19.	Palacios et al., 2018 (96)	USA	Healthy adults	51	Household food purchasing behavior, three 24-hr recall, FFQ	8 weeks	“MyNutriCart” app didn't led to significant improvements in food related behavior compared to traditional group
20.	Recio-Rodriguez et al., 2016 (85)	Spain	Healthy adults	833	Mediterranean Diet Adherence Screener questionnaire.	3 months	No difference was found on adherence to Mediterranean diet
21.	Sandborg et al., 2021 (36)	Sweden	Overweight pregnant moms	305	3-day dietary record using Swedish Healthy Eating Index	6 months	Smartphone App (HealthyMoms) promoted healthy dietary behaviors.
22.	Turner-McGrievy and Tate, 2011 (97)	USA	Overweight adults	96	2 unannounced days of dietary intake	6 months	Days/week of reported diet monitoring did not differ between Podcast + Mobile and podcast-only groups
23.	Weber, 2021 (98)	Germany	Healthy Adults	332	Sustainable food choice/3-item scale	Not clear	The using of mobile devices reduces decision uncertainty and increases sustainable food choices.
24.	Wengreen et al., 2021 (87)	USA	Children	1,859	Photo estimates of fruit and vegetable consumption	3 months	Children attending the FIT Game schools consumed more fruit and vegetables
25.	Whitelock et al., 2019 (99)	UK	Healthy adults	107	Three Factor Eating Questionnaire-21	8 weeks	No difference in self-reported eating behavior across groups.

FFQ, food frequency questionnaire; IDQ, index of diet quality; IES-1, intuitive eating scale; TFEQ-R18, three-factor eating questionnaire.

### The effect of web-based mobile app interventions on healthy eating behavior

Out of the 34 studies included in the final narrative synthesis, 16 focused on the effects of web-based mobile applications on healthy eating behavior. These studies assessed behaviors such as reducing sugar and fat intake, increasing fruit and vegetable consumption, decreasing salt intake, and adherence to the Mediterranean diet. It was found that using social media or internet-connected nutrition apps, which allow users to share thoughts and receive personalized expert advice, encouraged the adoption of healthier eating habits.

Web-based smartphone apps promote healthy eating behaviors in individuals with chronic diseases. For example, apps like Healthwatch 360 and DHealth Bar (a WeChat applet) improve the eating behavior of cancer and diabetic patients. In cardiac patients, it increases adherence to the Mediterranean diet (100–103).

Internet-based mobile apps have had a significant impact on promoting desired feeding practices in pregnant women and children. For example, a study by Billah et al. involving 1,500 participants showed a 16% increase in exclusive breastfeeding and improved dietary diversity scores among children in the intervention group (104). Another study by Van Dijk et al. focused on pregnant women and found that smartphone-based interventions increased the consumption of fruits and vegetables while reducing the intake of unhealthy snacks (105).

Online mobile apps have had a positive impact on the eating habits of both healthy adolescents and adults. One study conducted in Italy, for instance, demonstrated that a two-week intervention utilizing text messages resulted in improved fruit and vegetable consumption among 623 participating adolescents (106). Moreover, Kaur et al. and Lim et al. conducted interventional studies on healthy and overweight adults, which demonstrated that SMART-eating effectively reduced fat, sugar, and salt intake while increasing fruit and vegetable consumption (105, 107) (Table 3).

**Table 3:** Summary of randomized controlled studies on the effect of internet-based mobile apps interventions on healthy eating behaviors, 2022.

S. N	Author (s), year	Study setting	Study population	Sample size	Outcome variable measurement method	Follow-up period	Main finding
1.	Ambeba et al., 2015 (108)	USA	Obese adults	210	Self-reported dietary intake	24 months	Daily, tailored feedback messages (DFB group), using mobile devices, may play an important role in the reduction of energy and fat intake as compared to the No-DFB group.
2.	Billah et al., 2022 (109)	Bangladesh	Pregnant mothers	1,500	EBF indicators and 24-hr recall	6 months	EBF was 16% higher in the intervention than the comparison arm at 4 months.
3.	Billah et al., 2022 (104)	Bangladesh	Children aged 6–23 months	1,500	Mean dietary diversity score using 24-hr recall	18 months	Mean dietary diversity score improved only for a short term but on older age.
4.	Carfora et al., 2016 (106)	Italy	Adolescents	634	Self-reported fruit and vegetable intake	2 weeks	Text messages can be used to increase FVI in adolescents.
5.	Chow et al., 2021 (100)	USA	Cancer survivors	41	FFQ -7-day, fitness and diet trackers	16 weeks	M-health was associated with greater reductions in targeted dietary factors and improvements in Healthy Eating Index-2015 score.
6.	Franco et al., 2022 (110)	UK	Healthy adults	438	eNutri FFQ and m-AHEI	12 weeks	The app improved short-term diet quality and increase engagement in healthy eating behaviors.
7.	Goni et al., 2020 (101)	Spain	Patients with Atrial Fibrillation	720	14-item Mediterranean Diet Adherence Screener (MEDAS)	2 years	Website and phone calls seems effective in increasing adherence to the Mediterranean diet
8.	Inauen et al., 2017 (111)	USA	Healthy adults	203	Daily servings of fruit/vegetables or unhealthy snacks	Not clear	Smartphone-based groups can promote fruit and vegetable consumption and decrease unhealthy snack intake.
9.	Kaur et al., 2020 (105)	India	Adults (index case) selected from family	732	Mean dietary intakes of fat, sugar, salt, fruit and vegetables	6 months	“SMART Eating” intervention was effective in reducing fat, sugar, and salt intake, and increased FVI
10.	Lim et al., 2021 (107)	Singapore	Overweight adults	190	Change in body weight, HbA1c, FBG, BP, lipids, and diet	6 months	The app intervention led to significant reductions in total energy, carbohydrate, sugar, total fat, and saturated fat intake.
11.	Van Dijk et al., 2020 (112)	Netherland	Pregnant women	254	Dietary Risk Score	24 weeks	SMART group had high compliance and improvements in vegetable intake
12.	Wu et al., 2020 (113)	China	Pregnant mothers	344	Exclusive breastfeeding rate	12 months	WeChat proved to be an effective method of promoting exclusive breastfeeding in early life.
13.	Xu, Zidu, et al., 2020 (102)	China	Individuals at high risk for type 2 DM	79	Self-report, anthropometric indices, FFQ-25	6 months	The intervention group significantly decreased energy, fat, and carbohydrate intake.
14.	Ahn et al., 2016 (103)	Korea	Diabetic patients	24	Nutrition knowledge, dietary attitude, eating behavior and diet intake	1 month	The program user group significantly improved dietary behaviors.

FVI, fruit and vegetable Intake; DFB, daily feedback based; AHEI, adult health eating index; FFQ, food frequency questionnaire.

## Discussion

The widespread availability and affordability of smartphones, along with the influence of social media, have harnessed the extensive capabilities of technology to assist individuals in improving their healthy eating habits. This study sought to evaluate the effectiveness of smartphone app-based interventions in promoting healthy dietary behaviors among diverse populations and in different countries. The findings of this study indicate that the use of smartphone and web apps has a significant impact on enhancing healthy eating behaviors. However, it is worth noting that in certain studies, changes in dietary behaviors were only maintained for a short duration.

This study included 39 interventional studies that used smartphone and web-based apps to provide information to diverse populations. The study involved healthy adults, overweight or obese adults, chronically ill patients, pregnant mothers, and children of different age groups.

Among the 14-smartphone app-based studies conducted on healthy adults (with the offline capability), the majority (10) did not show a positive impact on modifying healthy eating behaviors. However, all four studies involving patients with chronic diseases indicated that the use of smartphone apps improved adherence to healthy dietary behaviors. Additionally, the findings regarding pregnant women and children showed mixed results. The hesitance of healthy adults to use smartphone apps can be attributed to the health belief model, which emphasizes the significance of factors such as perceived susceptibility, perceived severity, and perceived benefits in promoting healthy behavioral changes (114). This suggests that policy engagement is still necessary to promote the adoption of health maintenance behaviors among healthy adults. In contrast, using smartphone apps to convey dietary messages had a significant positive impact on the eating behavior of patients with chronic diseases. Altering one's eating habits is crucial for effectively managing chronic diseases. Patients are well aware of the negative consequences that can arise from making irresponsible food choices. Moreover, smartphone apps designed to help reduce salt or sodium consumption can serve as a platform to achieve the WHO's goal of reducing the global population's sodium intake by 30% by 2025 (115). The finding regarding the role of smartphone app-based interventions in healthy adults aligns with previous systematic reviews (12, 29). However, this finding contradicted a review of trials involving overweight adults. The review found that the use of smartphone apps improved adherence to diets that included lower-calorie, low-fat, and high-fiber foods (116). The discrepancy may be attributed to variations in the scope of the reviews, eligibility criteria for study selection, and the number of studies included in the review, all of which can impact the conclusions drawn. The effects of smartphone app-based dietary interventions on both healthy and overweight adults were inconsistent in the studies included. It is worth noting that these inconsistencies were not due to variations in sample size, intervention duration, or study settings. These findings underscore the significance of carefully identifying the target consumers when designing smartphone app-based interventions. The main finding from all 14 studies was that web-based mobile apps effectively encouraged the development of healthy eating habits in various groups of people, such as healthy adults, overweight or obese adults, pregnant women, and individuals with chronic diseases. The positive outcome can be attributed to intervention methods that involved receiving regular messages and feedback from professionals. Additionally, the use of educational videos was effective in conveying information. These strategies successfully engaged participants and facilitated changes in their healthy eating behaviors. Visual content, such as images and videos, is highly appealing to consumers, particularly in the current age of social media platforms like TikTok. As consumers have limited time and patience for reading lengthy texts, they prefer information that engages multiple senses and is presented in a concise format (117). A meta-analysis study also indicated that using pictorial health information significantly increases knowledge and understanding, especially for populations with lower health literacy. Furthermore, the use of icons with minimal accompanying words is found to be highly effective in conveying health information (118). Furthermore, our study emphasized the importance of not just the presentation format of the message but also the consistency and professional feedback provided through online platforms as vital elements of web-based dietary interventions. Our findings align with a study that demonstrates the significant positive impact of using web-based apps on weight loss and calorie reduction. Furthermore, the study highlights that tailored interventions are considerably more effective than non-tailored interventions (20). Our results are consistent with a previous review that highlighted the significance of integrating digital-based interventions with personalized feedback and counseling to achieve long-lasting, desired dietary behavior (45). Furthermore, this study aligns with a review that highlights the efficacy of digital technology, particularly apps, in improving stakeholder relationships within the agro-food chain and advancing urban and regional food systems (119). The result can be better elucidated by considering the concepts of social and behavioral change communication (SBCC) and health belief models (HBM). According to these models, individuals require repetitive communication through channels that are suitable and preferred within their community to effectively promote desired changes in behavior (120). Meanwhile, the results of our study have global implications for improving countries' efforts to expand internet infrastructure, making smartphones more affordable, and promoting digital literacy. These combined efforts will enhance the effectiveness of public-health dietary interventions.

The strength of this study lies in its inclusion of only randomized controlled trials and the assessment of article quality using the Cochrane risk-of-bias tool. This enhances the credibility of the results. However, this review has some limitations. The extensive scope of this study, which includes a wide range of study populations and dietary interventions, hinders the ability to carry out a meta-analysis. Furthermore, the lack of studies conducted in low-income countries, the disparities in digital access both within and between affluent countries, and the increasing prices of healthy food could impact the generalizability of the findings.

## Conclusion

In general, the use of smartphone apps (offline or web-based) has led to positive changes in healthy eating habits among different populations and individuals with varying health conditions. Both platforms of smartphone apps contribute to promoting healthy dietary behaviors among patients with chronic diseases. Similarly, the impact of tailored messages, along with professional feedback through web-based app platforms, on promoting public health dietary interventions has been significant. However, there is no consistent reporting on the effectiveness of non-internet-based smartphone apps on the dietary behavior of healthy adults.

### Recommendation

The use of mobile apps, including social media, to deliver public health dietary interventions should be a top policy priority. Furthermore, it is important to identify target consumers before developing digital technologies, as their effectiveness can vary among different populations. Continued efforts are needed to improve smartphone and internet accessibility for a wider range of population groups. It is recommended to conduct more large-scale randomized controlled trials (RCTs) in low- and middle-income countries to gather more comprehensive evidence. Meta-analysis studies that examine specific dietary interventions in single population groups are also recommended. Further studies that specifically target healthy adults and adolescents are necessary.
